# Maternal iron metabolism gene variants modify umbilical cord blood lead levels by gene-environment interaction: a birth cohort study

**DOI:** 10.1186/1476-069X-13-77

**Published:** 2014-10-06

**Authors:** Mateusz P Karwowski, Allan C Just, David C Bellinger, Rebecca Jim, Earl L Hatley, Adrienne S Ettinger, Howard Hu, Robert O Wright

**Affiliations:** Division of General Pediatrics, Pediatric Environmental Health Center, Boston Children’s Hospital, Boston, MA USA; Department of Environmental Health, Harvard School of Public Health, Boston, MA USA; Department of Neurology, Boston Children’s Hospital, Boston, MA USA; Local Environmental Action Demanded Agency, Inc, Miami, OK USA; Department of Chronic Disease Epidemiology, Center for Perinatal, Pediatric, and Environmental Epidemiology, Yale School of Public Health, New Haven, CT USA; Divisions of Clinical Public Health, Epidemiology, Global Health, and Occupational and Environmental Health, Dalla Lana School of Public Health, University of Toronto, Toronto, Ontario Canada; Departments of Preventive Medicine and Pediatrics, Icahn School of Medicine at Mt. Sinai, New York, NY USA

**Keywords:** Hemochromatosis gene, *C282Y*, *H63D*, Lead, Pediatric, Polymorphism, Prenatal, *P570S*, Transferrin gene

## Abstract

**Background:**

Given the relationship between iron metabolism and lead toxicokinetics, we hypothesized that polymorphisms in iron metabolism genes might modify maternal-fetal lead transfer. The objective of this study was to determine whether maternal and/or infant transferrin (*TF*) and hemochromatosis (*HFE*) gene missense variants modify the association between maternal blood lead (MBL) and umbilical cord blood lead (UCBL).

**Methods:**

We studied 476 mother-infant pairs whose archived blood specimens were genotyped for *TF P570S*, *HFE H63D* and *HFE C282Y*. MBL and UCBL were collected within 12 hours of delivery. Linear regression models were used to examine the association between log-transformed MBL and UCBL, examine for confounding and collinearity, and explore gene-environment interactions.

**Results:**

The geometric mean MBL was 0.61 μg/dL (range 0.03, 3.2) and UCBL 0.42 (<0.02, 3.9). Gene variants were common with carrier frequencies ranging from 12-31%; all were in Hardy-Weinberg equilibrium. In an adjusted linear regression model, log MBL was associated with log UCBL (β = 0.92, 95% CI: 0.82, 1.03; p < 0.01) such that a 1% increase in MBL was associated with a 0.92% increase in UCBL among infants born to wild-type mothers. In infants born to *C282Y* variants, however, a 1% increase in MBL is predicted to increase UCBL 0.65% (β_Main Effect_ = −0.002, 95% CI: −0.09, −0.09; p = 0.97; β_Interaction_ = −0.27, 95% CI: −0.52, −0.01; p = 0.04), representing a 35% lower placental lead transfer among women with MBL 5 μg/dL.

**Conclusions:**

Maternal *HFE C282Y* gene variant status is associated with greater reductions in placental transfer of lead as MBL increases. The inclusion of gene-environment interaction in risk assessment models may improve efforts to safeguard vulnerable populations.

**Electronic supplementary material:**

The online version of this article (doi:10.1186/1476-069X-13-77) contains supplementary material, which is available to authorized users.

## Background

Nearly 1% of women aged 20–39 living in the United States have a blood lead concentration (BPb) greater than or equal to 5 μg/dL (n = 420,000) [1, 2]. Due to lead transfer across the placenta, their children are at risk of being born with BPb above the CDC reference level of 5 μg/dL [3] and are being exposed to a neurotoxicant, for which no threshold for harmful effects has been identified [4], at a period of heightened vulnerability [5, 6].

Prenatal lead exposure is linked to spontaneous abortion [7], low birth weight [8], preterm birth [9], small for gestational age birth [10], congenital malformation [8, 11], and deficits in intellectual development [12–15]. The transfer of lead across the placenta from mother to fetus is complex and incompletely understood, but is the sole factor determining fetal exposure. Sources of maternal lead can be extrinsic (maternal environmental exposure) or intrinsic (via mobilization of lead in bone stores).

The mechanisms for placental uptake and efflux of lead are not well defined, but likely include metal-specific proteins and receptors common to iron metabolism. Hence, genes that regulate iron metabolism may play an important role in placental lead transfer. Transferrin (*TF*) and hemochromatosis (*HFE*) are two iron metabolism genes for which functional variants are prevalent in North America. The *TF* gene is located on 3q22.1 and codes for the glycoprotein transferrin, which is responsible for intercellular iron transport [16]. While the *TF* gene is highly polymorphic, a common variant is the *P570S* missense single nucleotide polymorphism (SNP) [17]. The prevalence of the *P570S* polymorphism is estimated at 15% in the US general population and 12.5% in Native Americans, though the latter is based on a small sample [17].

The *HFE* gene is located on 6p21.3 and codes for the HFE protein, which regulates iron cellular uptake by binding transferrin and decreasing activation of transferrin receptors, thereby reducing transport across cellular membranes [18]. HFE protein influences the expression of other metal transporters found in the intestinal tract such as divalent metal transporter 1 (DMT1) [19, 20]. Two missense SNPs (*H63D* and *C282Y*) in the *HFE* gene are overrepresented among individuals with the hemochromatosis phenotype in which iron is hyperabsorbed. In a large screening study based in North America, 24% of whites and 20% of Native Americans carried variants for *H63D*, while 10% of whites and 5% of Native Americans carried variants for *C282Y*[21].

Though iron metabolism genes play a role in lead metabolism and toxicity among children and adults [22–29], it remains unclear how normal adaptations in homeostatic function and physiology during pregnancy may complicate the relationship between iron metabolism genes, maternal BPb, and maternal-fetal lead transfer. The objective of this study is to determine whether genomic variations in maternal and/or infant *TF* and *HFE* genes modify the association between maternal and umbilical cord blood lead.

## Methods

### Study participants

Subjects were enrolled in a prospective birth cohort study based around the Tar Creek Superfund Site (TCSS) in northeastern Oklahoma. The site is a former lead and zinc mega-mine that was in operation from 1891-1970s [30]. Lead, cadmium and manganese are among the toxic metals that contaminate its estimated 50 square miles. As part of a collaborative effort between the Harvard School of Public Health (HSPH; Boston, MA, USA), a community-based non-profit – Local Environmental Action Demanded Agency (LEAD; Miami, OK, USA), and Integris Baptist Regional Health Center (IBRHC; Oklahoma City, OK, USA), a birth cohort was established in 2002 to study early life exposure to metals, psychosocial stress, and their interactions on neurocognitive development. The Institutional Review Boards at HSPH and IBRHC approved the research protocol.

Between 2002 and 2007, 713 pregnant women presenting for prenatal care or delivery to the only hospital in Ottawa County (Integris Baptist Regional Health Center; Miami, OK, USA) were enrolled in the cohort. Eligibility criteria were: 1) giving birth at the Integris Hospital in Miami, OK; 2) intention to reside in the area for the following two years; 3) not already enrolled in the study with another child; and 4) being proficient in English. After confirming study eligibility and providing informed consent, participants provided demographic, medical and environmental information via standardized, interviewer-administered questionnaires. Delivery room staff gathered anthropometric measures on newborns within twelve hours of birth. Deoxyribonucleic acid (DNA) was extracted on a subset of the first 500 children born. We limited our analysis to the 476 singleton pregnancies in which both mother and infant were successfully genotyped for the SNPs of interest.

### Blood measurements

Umbilical cord and maternal venous whole blood was collected in trace element-free tubes (BD Vacutainer® reference number 368380; Becton Dickinson, Franklin Lakes, NJ, USA) and tubes for DNA extraction (PAXgene® Blood DNA Tubes; PreAnalytiX GmbH, Hombrechtikon, Switzerland) within twelve hours of delivery. Samples were frozen and subsequently shipped in batches to the Trace Metals Laboratory at HSPH for analysis. All sample preparation and handling occurred in a Class 10,000 clean room (ISO 7), under a Class 100 clean hood (ISO 5). One gram of whole blood from each sample was digested in 1 mL of concentrated nitric acid for 24 hours. After adding 0.5 mL of 30% hydrogen peroxide, the samples were diluted with deionized water to a final volume of 10 mL. Blood lead was measured using an inductively-coupled plasma mass spectrometer (ICP-MS, Elan 6100; Perkin Elmer, Norwalk, CT, USA).

Quality control measures included analysis of initial and continuous calibration verification standards, procedural blanks, duplicate samples, and spiked samples. Comparisons were drawn from National Institute of Standards and Technology Standard Reference Material for trace elements in water (NIST SRM 1643d) and lead in blood (NIST SRM 955b). The average of five replicate measurements was reported as the final value. The lower limit of detection (LOD) was 0.02 micrograms lead per deciliter whole blood.

### Transferrin (*TF*) and hemochromatosis (*HFE*) genotyping

High-molecular-weight DNA was extracted from white blood cells of archived umbilical cord blood with commercially available PureGene Kits (Gentra Systems, Minneapolis, MN, USA). After DNA quantification, samples were adjusted to TE (Tris-EDTA) buffer, partitioned into aliquots, and stored at −80°C. Multiplex polymerase chain reaction assays were designed using Sequenom SpectroDESIGNER software by inputting sequence containing the SNP site and 100 base pairs of flanking sequence on either side of the SNP. Three SNPs were multiplexed: (*TF*) *P570S* (rs1049296), (*HFE*) *H63D* (rs1799945), and (*HFE*) *C282Y* (rs1800562). The extension product was then spotted onto a 384 well spectroCHIP before being flown in the MALDI-TOF mass spectrometer.

### Potential confounders

While covariates that are extrinsic to the maternal-fetal system, such as socioeconomic status, would not theoretically confound the relationship between maternal and umbilical cord blood lead, factors that are intrinsic to this system may do just that. A woman’s iron status, for example, is associated with her iron metabolism genotype and may also impact placental lead transfer by influencing the expression of metal transport machinery that is common to both iron and lead.

Regardless of theoretical plausibility, we assessed several covariates for potential confounding of the association between maternal and umbilical cord blood lead. Infant gestational age, birth weight, and gender were considered in a sensitivity analysis, as were maternal race, ethnicity, and socioeconomic status. Adjustments for anemia and iron status were also made using the covariates for which maximum data were available: serum ferritin in infants, and hematocrit in both infants and mothers.

### Statistical analysis

We examined summary statistics and distributional plots for all variables prior to analysis. Two blood lead values that were reported as negative values were converted to the instrument LOD divided by the square root of two. We calculated the arithmetic and geometric means and standard deviations of blood lead levels stratified by genotype (wild-type versus carrier), testing for significance using Student’s *t*-test for independent samples. We inspected the distributions of *TF* and *HFE* genotypes and tested their frequencies for deviation from the Hardy-Weinberg principle using Pearson’s chi-square test. Given that the allele frequencies were relatively low, we chose dominant genetic models for each allele, combining heterozygotes and homozygote variants into a single indicator term.

Blood lead levels followed a log normal distribution and were transformed in order to satisfy the normality condition for regression analysis. As a result, beta coefficients from regression models represent the percent change in UCBL for a 1% change in MBL. An *a priori* type I error rate (α) of 0.05 was set for all tests.

We first explored the relationship between umbilical cord and maternal venous blood lead through a bivariate analysis limited to mother-infant pairs in which both mother and infant were wild-type (*n* = 159). The linear relationship between these two variables informed our choice to analyze the full dataset using linear models. Next, we re-ran bivariate models using the entire cohort (*n* = 476) and evaluated for potential confounding using the aforementioned independent variables. Confounders were included in subsequent regression models if they changed the parameter estimate for the main effect by at least 10%.

We tested for effect modification by genotype and included interaction terms for all combinations of maternal and infant genotype and maternal blood lead. Separate models were built for infants and mothers in order to assess each group independently. Lastly, infant and maternal interaction terms were combined into a final multivariable regression model. Given the potential for collinearity between maternal and infant genotype in this final model, correlations between independent variables and collinearity diagnostics were examined; an *a priori* variance inflation factor threshold was set at 10. Regression diagnostics were run on all models.

Data were analyzed using SPSS Statistics for Windows, Version 19.0 (IBM Corporation, Armonk, NY, USA) and R Version 2.15.3 (R Foundation for Statistical Computing, Vienna, Austria).

## Results

Descriptive characteristics for mothers and infants from the original birth cohort are listed in Table 1, stratified by the availability of genotype information. Our study cohort is limited to the 476 mother-infant pairs in whom blood lead levels and genotype information was determined. The mean weight, length, and head circumference of neonates corresponded to World Health Organization international growth charts, which are recommended by the CDC for use in children up to age 24 months [31].

**Table 1:** **Characteristics of mother-infant pairs from Ottawa County, OK, stratified by availability of genotype information** ^**a**^

Characteristic	Complete genotype information		Unavailable genotype information	
	***n*** = 476	Descriptive measure ^b^	***n*** = 232	Descriptive measure ^b^
**Mothers**				
Age at birth of infant (years)	476	24.5 (5.5)	228	24.6 (5.2)
Race/Ethnicity^c^				
White	341	70%	185	79%
Native American	120	25%	65	28%
Hispanic	24	5%	4	1.7%
Asian	16	3.3%	3	1.3%
African American	4	0.8%	1	0.4%
Education				
< 12th grade	118	25%	66	28%
≥ 12th grade	358	75%	166	72%
Hematocrit at delivery (%)	468	34.3 (3.8)	225	34 (3.4)
Hematocrit at 28 weeks gestation (%)	409	35.5 (3.4)	197	34.9 (3.3)
Blood lead at delivery (μg/dL)^d^	476	0.61 (1.87)	183	0.54 (1.7)
**Infants**				
Gender (% female)	475	45%	230	48%
Gestational age (weeks)	473	39.2 (1.3)	229	38.8 (1.5)
Birth weight (g)	476	3385 (485)	229	3300 (438)
Birth length (cm)	464	49.8 (3)	227	50.4 (2.4)
Birth head circumference (cm)	458	34.7 (2.3)	225	34.3 (2)
Hematocrit at birth (%)	132	55.2 (7.8)	225	55.9 (7.2)
Serum ferritin at birth (ng/mL)	244	154 (92)	177	156 (97)
Umbilical cord blood lead (μg/dL)^d^	476	0.42 (2.12)	180	0.38 (1.8)

^a^Complete data are missing for certain characteristics; ^b^Arithmetic mean ± SD or% as indicated; ^c^Overlap between categories exists because individuals could self-identify as belonging to two or more races; ^d^Geometric mean ± GSD.

Genotype frequencies for all 476 mother-infant pairs were successfully determined for all three SNPs. The genotype frequencies for mothers and infants are shown in Tables 2 and 3, respectively; all were found to be in Hardy-Weinberg equilibrium (α = 0.05). There were no statistically significant differences between geometric mean blood lead values when stratified by variant status.

**Table 2:** **Maternal genotype frequencies and geometric mean blood lead levels for dominant models** ^**a,b**^

Single-nucleotide polymorphism	No.	Genotype frequencies	Dominant modeling	Geometric mean BLL ^c^ (95% CI ^d^ )
*TF P570S* (rs1049296)				
Homozygous wild-type	355	CC = 0.75	wild-type	0.62 (0.58, 0.66)
Heterozygous	109	CT = 0.23	variant	0.60 (0.55, 0.67)
Homozygous variant	12	TT = 0.02		
*HFE H63D* (rs1799945)				
Homozygous wild-type	350	HH = 0.74	wild-type	0.61 (0.57, 0.65)
Heterozygous	117	HD = 0.24	variant	0.62 (0.56, 0.69)
Homozygous variant	9	DD = 0.02		
*HFE C282Y* (rs1800562)				
Homozygous wild-type	417	CC = 0.88	wild-type	0.62 (0.58, 0.65)
Heterozygous	55	CY = 0.11	variant	0.60 (0.51, 0.70)
Homozygous variant	4	YY = 0.01		

^a^All genotype frequencies are in Hardy-Weinberg equilibrium (α = 0.05); ^b^None of the differences in geometric mean BLL by variant status are statistically significant; ^c^blood lead level (μg/dL); ^d^confidence interval.

**Table 3:** **Infant genotype frequencies and geometric mean umbilical cord blood lead levels for dominant models** ^**a,b**^

Single-nucleotide polymorphism	No.	Genotype frequencies	Dominant modeling	Geometric mean BLL ^c^ (95% CI ^d^ )
*TF P570S* (rs1049296)				
Homozygous wild-type	338	CC = 0.71	wild-type	0.43 (0.40, 0.47)
Heterozygous	129	CT = 0.27	variant	0.40 (0.36, 0.46)
Homozygous variant	9	TT = 0.02		
*HFE H63D* (rs1799945)				
Homozygous wild-type	330	HH = 0.69	wild-type	0.44 (0.40, 0.48)
Heterozygous	132	HD = 0.28	variant	0.39 (0.35, 0.44)
Homozygous variant	14	DD = 0.03		
*HFE C282Y* (rs1800562)				
Homozygous wild-type	415	CC = 0.87	wild-type	0.42 (0.39, 0.46)
Heterozygous	58	CY = 0.12	variant	0.43 (0.36, 0.51)
Homozygous variant	3	YY = 0.01		

^a^All genotype frequencies are in Hardy-Weinberg equilibrium (α = 0.05); ^b^None of the differences in geometric mean BLL by variant status are statistically significant; ^c^blood lead level (μg/dL); ^d^confidence interval.

In a bivariate analysis restricted to wild-type mother-infant pairs (*n* = 157), MBL was linearly associated with UCBL (β = 0.79; 95% CI 0.69, 0.88; *p* < 0.001). When this analysis was repeated for the log-transformed values, the correlation between these two variables strengthened (β = 0.99; 95% CI 0.88, 1.10; *p* < 0.001). Similar parameter estimates were obtained when the bivariate analysis was expanded to include all 476 mother-infant dyads (data not shown).

In our sensitivity analysis, the association between maternal and umbilical cord blood lead was not confounded by gestational age, birth weight, infant gender, maternal race/ethnicity, socioeconomic status, and infant hematocrit or serum ferritin. While the parameter estimate for maternal hematocrit at delivery was statistically significant (β = −0.008; 95% CI −0.01, −0.003; *p* < 0.01), it did not meaningfully change the beta estimate for MBL (β_adjusted_ = 0.84; 95% CI 0.77, 0.92; *p* < 0.001; β_unadjusted_ = 0.85; 95% CI 0.78, 0.93; *p* < 0.001). Therefore, it did not satisfy the *a priori* definition for confounding.

Lead-hematocrit interaction terms were also modeled under the premise that hematocrit might modify placental lead transfer if, for example, varying amounts of lead cross the placenta in the presence of low or normal hematocrit. The interaction between MBL and both maternal hematocrit at delivery (β_Interaction_ = −0.02; 95% CI −0.04, 0.001; *p* = 0.06), as well as maternal hematocrit at 28 weeks (β_Interaction_ = −0.02; 95% CI −0.05, 0.003; *p* = 0.08) were marginally significant. While the interaction between MBL and umbilical cord hematocrit at birth (β_Interaction_ = −0.03; 95% CI −0.04, −0.01; *p* = 0.001) was statistically significant, this result should be interpreted with caution; since umbilical cord hematocrit was not routinely measured, the sample available for analysis was limited to only 132 of the original 476 study participants.

Lastly, we explored the potential for a gene-environment interaction between iron metabolism genotype and MBL by regressing log-transformed values of UCBL on similarly-transformed values of MBL. In order to account for potential confounding between genotype variants, the main effect terms for maternal and infant *TF* and *HFE* variant status, along with their respective interaction with MBL, were modeled together. Though infant and maternal variant status were modestly correlated for each gene variant (Spearman ρ ranging from 0.43 to 0.52), variance inflation factors did not exceed 3.4 and thus raised little suspicion for collinearity. This conclusion was strengthened by two additional observations: 1) parameter estimates remained stable when potentially collinear variables were selectively excluded from the full model described in Table 4 and 2) in regression models that were limited to either infant or maternal gene variants (Additional files 1 and 2, respectively), the main effect and interaction term parameter estimates were very similar to those from the full regression model.

**Table 4:** **Multivariable regression model of iron metabolism gene variants, log-transformed maternal blood lead, and their interaction on log-transformed umbilical cord blood lead (** ***n =*** **476)** ^**a,b,c**^

Predictor	β	95% CI	P-value
Constant	−0.17	−0.21, −0.13	**<0.01**
Maternal Blood Lead (MBL)	0.92	0.82, 1.03	**<0.01**
Maternal *TF P570S*	0.04	−0.03, 0.11	0.28
MBL × Maternal *TF P570S*	−0.13	−0.35, 0.09	0.24
Maternal *HFE H63D*	−0.07	−0.14, 0.01	0.08
MBL × Maternal *HFE H63D*	−0.12	−0.34, 0.11	0.32
Maternal *HFE C282Y*	−0.002	−0.09, 0.09	0.97
MBL × Maternal *HFE C282Y*	−0.27	−0.52, −0.01	**0.04**
Infant *TF P570S*	−0.06	−0.13, 0.003	0.06
MBL × Infant *TF P570S*	−0.03	−0.24, 0.18	0.81
Infant *HFE H63D*	0.01	−0.06, 0.08	0.77
MBL × Infant *HFE H63D*	0.10	−0.11, 0.32	0.34
Infant *HFE C282Y*	−0.02	−0.11, 0.07	0.65
MBL × Infant *HFE C282Y*	−0.05	−0.28, 0.17	0.64

^a^Blood lead concentrations are log-transformed, beta coefficients are therefore interpreted as percent change in umbilical cord blood lead for a 1% change in maternal blood lead; ^b^Collinearity diagnostics indicate a maximum variance inflation factor of 3.4; ^c^R^2^ for the model equals 0.53 and adjusted R^2^ equals 0.52; ^d^bolded values are statistically significant at α = 0.05.

**Figure 1:**
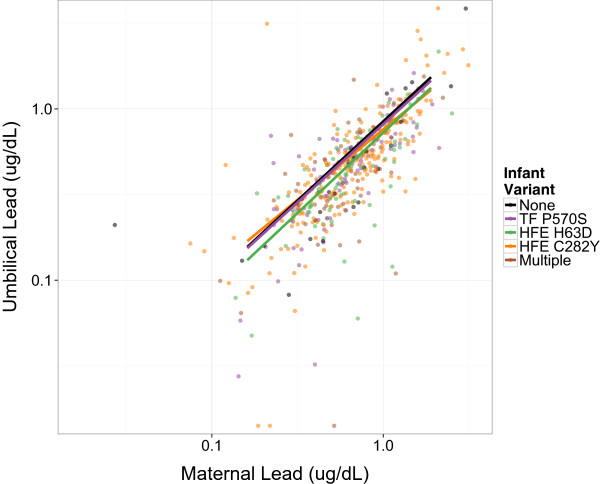
**Modification by** ***infant*** **genotype of the association between maternal and umbilical cord blood lead.** Adjusted for maternal genotype. Three values ≤0.005 ug/dL for umbilical lead not shown.

**Figure 2:**
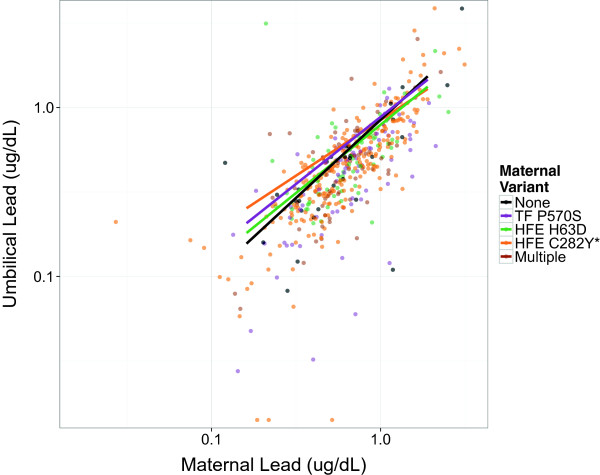
**Modification by** ***maternal*** **genotype of the association between maternal and umbilical cord blood lead.** *p < 0.05. Adjusted for infant genotype. Three values ≤0.005 ug/dL for umbilical lead not shown.

**Figure 3:**
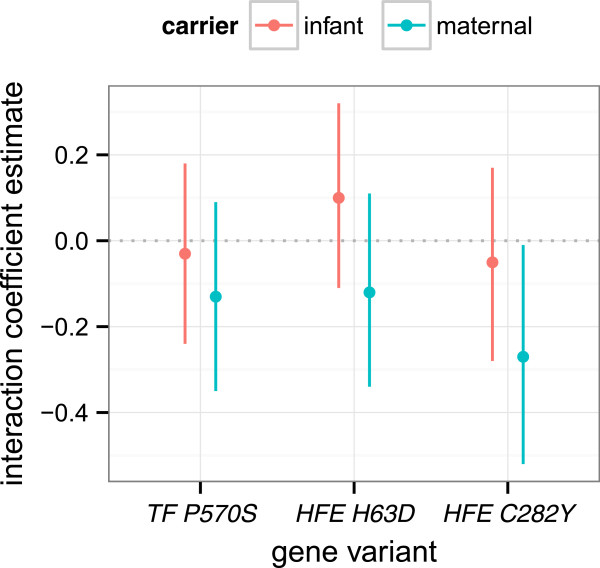
**Gene-environment interaction coefficient estimates from a model regressing umbilical cord on maternal blood lead.**

In a fully-adjusted model accounting for infant and maternal iron metabolism genotype and gene-environment interaction terms, MBL retained its association with UCBL (β = 0.92; 95% CI 0.82, 1.03; *p* < 0.001; Table 4). Among wild-type mother-infant pairs, a 1% increase in MBL is expected to increase UCBL by 0.92%. The association between UCBL and MBL was not modified by infant gene variant status or infant genotype-MBL interaction terms (Figure 1). Likewise, maternal gene variant main effect terms were not significant. However, the interaction between maternal *HFE C282Y* genotype and MBL was significant (β_Interaction_ = −0.27; 95% CI −0.52, −0.01; *p* = 0.04; Table 4) and attenuated the relationship between UCBL and MBL (Figure 2). While the other two maternal genotype interaction terms were not statistically significant, all three consistently trended towards a protective effect on UCBL (Figure 3). Notably, these results did not differ appreciably from output generated by the two regression models that were limited to either infant or maternal gene variants (see Additional files 1 and 2, respectively).

Among infants born to mothers who have the *HFE C282Y* variant, a 1% increase in MBL is predicted to increase UCBL by 0.65%, as opposed to 0.92% for infants born to mothers who are wild-type. Factoring the log transformation, our model predicts that for two pregnant women with BPb 5 μg/dL but differing *HFE C282Y* genotypes, UCBL will be 3 μg/dL (95% CI 2.3, 3.9) in the infant born to the wild-type mother and 1.95 μg/dL (95% CI 0.8, 4.7) in the infant born to the mother carrying the *HFE C282Y* variant.

## Discussion

The results of this study suggest that maternal iron metabolism genotype is a modifier of placental lead transfer. Infants born to women with the *HFE C282Y* gene variant have lower umbilical cord blood lead concentrations. Further, the attenuating impact of this gene-environment interaction on placental lead transfer strengthens as MBL rises. While MBL seems to be the key determinant of UCBL, maternal iron metabolism gene variants modify this relationship in a manner that is potentially clinically significant in exposed populations.

Additional results suggest that *fetal* iron metabolism gene variant status did not modify the relationship between UCBL and MBL. In other words, it appears that lead transfer across the placenta is not altered significantly by fetal iron metabolism genes, but primarily by maternal genotype. This finding has direct relevance for explaining the biology that underlies placental lead transfer.

At least four polymorphic genes are recognized to influence lead toxicokinetics and toxicodynamics: delta-aminolevulinic acid dehydratase (*ALAD*), vitamin D receptor (*VDR*), *TF* and *HFE*[22, 32]. Genetic variability may influence bioavailability and susceptibility in a complex fashion, interrelating with other individual characteristics such as age and micronutrient status to produce differential outcomes. For example, studies have shown that while elderly men who carry *HFE* gene variants have lower tibia, patella, and blood lead concentrations than wild-type subjects [23], children who carry either *HFE* or *TF* variants have higher BPb than wild-type peers [22]. Moreover, the impact of iron metabolism gene variants on lead metabolism seems to be amplified in the presence of multiple genotypic variant alleles, akin to a dose–response relationship [22, 24, 25].

While the role of iron metabolism gene variants in primary lead exposure and bioaccumulation has been studied, relatively little is known about how maternal and fetal genetic polymorphisms affect lead transfer across the placenta. This study adds to the existing literature by providing evidence that fetal lead exposure is attenuated among children born to mothers who carry variants for maternal iron metabolism genes.

The strong association between umbilical cord and maternal blood lead seen in this cohort has been observed in many studies [33–36]. However, the toxicokinetics of placental lead transfer are not well understood. Lead uptake by the placenta occurs passively [37] and is likely influenced by several proteins including, for example: HFE protein, which is expressed on the apical plasma membrane of placental syncytiotrophoblasts near the maternal circulation, and which interacts with transferrin to regulate cellular iron absorption [38]; DMT1, an iron transport protein that is also found in syncytiotrophoblasts and other tissues, and that plays a vital role in iron homeostasis [39–42]; and metallothioneins, which are small, cysteine-rich proteins found in abundance in placental tissue, have high metal binding properties, and participate in metal storage and detoxification [43–45]. Less is known about mechanisms underlying lead efflux [5]. Though the basolateral iron exporter ferroportin seems to be the sole cellular iron transporter [46], it may or may not play a role in lead transport.

Placental iron transfer occurs at the apical plasma membrane of syncytiotrophoblasts via transferrin receptor-mediated endocytosis. Since HFE protein is expressed at this site and is physically associated with the transferrin receptor, it has been hypothesized that HFE protein regulates the transfer of iron from maternal to fetal circulation [38]. While wild-type HFE protein binds to β_2_ microglobulin and is transported to the cell surface where it interacts with transferrin to regulate iron, these normal functions are altered in individuals who carry the *HFE C282Y* gene mutation. Given that lead is carried by these same iron transporters and receptors, it is possible that functional perturbations in the HFE protein resulting from *HFE C282Y* gene mutations may modify placental lead transfer.

Other hypotheses may also explain the biologic basis for decreased placental lead transfer among women who carry iron metabolism gene variants. For example, these women are more likely to have heightened pre-pregnancy stores of iron, which may decrease expression of metal-specific transporters or proteins and thereby lessen gastrointestinal lead absorption. Another potential explanation is the creation of a lead-sink in which lead moves freely into placental tissues via up-regulation of metal transport mechanisms [26] but is slow to exit into the fetal circulation due to changes in expression of proteins that bind lead. Perhaps by saturating these transporters, higher maternal levels of micronutrients – specifically zinc and manganese –reduce lead transfer to the fetus [34].

Other research in this area has shown that genetic polymorphisms in iron metabolism genes not only modify lead kinetics, but also confer variable host response to lead exposure. Carriers of the *TF P570S* gene variant, for example, lose significantly more IQ points per unit of blood lead than children with wild-type genotypes [27]. Lead-related decreases in birth weight [28], advancement of cognitive decline [24], widening of pulse pressure [29], and prolongation of the QT-interval [25] have been shown to be amplified in *HFE* gene variant carriers.

This study has several limitations. Whereas lead toxicokinetics are likely influenced by an array of functional gene polymorphisms, such as those involved in calcium regulation, we investigated only a few iron-specific candidate genes. Other genetic variants may modify placental transfer as well, but we did not have sufficient information in our dataset to examine them. Additionally, genes in linkage disequilibrium with the *HFE* gene might explain our results. Such confounding is unlikely, however, because other known iron transport genes are not located in this genomic region and *HFE C282Y* is a well-characterized functional variant.

Though it is characteristic of the US population, the narrow range of exposure among women in this study limits our ability to extrapolate findings to populations that have higher average blood lead levels. While residual confounding is possible, any environmental factor that confounded our findings would have to be associated with both placental lead transfer and *HFE* genotype. Apart from micronutrient status of iron and calcium, for example, it seems unlikely that there are environmental factors that could be associated with both placental lead transfer and *HFE* genotype. Introducing additional biomarkers of iron and calcium sufficiency might clarify the influence of maternal and fetal micronutrient status on placental lead transfer.

Statistical considerations include the possibility that the significant interaction between MBL and maternal *HFE C282Y* may be due to a Type I error related to multiple testing. Conversely, a Type II error due to insufficient power may explain why the remaining gene-environment interaction parameter estimates were no different from the null. Finally, like all new findings, these results require validation in other cohorts.

When taken in the context of current trends in clinical medicine and population health, the results of this study have interesting implications. Identifying factors that modify placental lead transfer might inform research in the emerging era of genomic medicine, where targeted therapeutics hold the promise of lessening the morbidity associated with prenatal lead exposure. Information about genetic modifiers of lead transfer may also inform how clinicians will advise, treat, and monitor their pregnant patients for micronutrient deficiencies. From a regulatory standpoint, results of this study prompt us to reconsider the current approach to risk assessment and ask whether methods should consistently address issues of genetic susceptibility.

## Conclusion

This study is among the first to investigate how maternal and fetal genetic polymorphisms secondarily affect lead transfer across the placenta. Through gene-environment interaction, maternal iron metabolism genotype appears to play a role in maternal-fetal lead transfer. We found that infants born to *mothers* with the *HFE C282Y* gene variant had lower umbilical cord blood lead levels relative to those born to women who were wild-type. Moreover, reductions in the proportion of maternal blood lead transferred across the placenta were greatest for infants whose mothers had higher blood lead levels. The incorporation of gene-environment information into risk assessment may improve regulatory efforts to safeguard the health of vulnerable populations.

## Electronic supplementary material

Additional file 1: **Parameter estimates from a multivariable regression model evaluating the effects of** ***infant*** **iron metabolism gene variants, log-transformed maternal blood lead, and their interaction on log-transformed umbilical cord blood lead.** (PDF 157 KB)

Additional file 2: **Parameter estimates from a multivariable regression model evaluating the effects of** ***maternal*** **iron metabolism gene variants, log-transformed maternal blood lead, and their interaction on log-transformed umbilical cord blood lead.** (PDF 72 KB)

## Abbreviations

*TF*: Transferrin gene [Reference Sequence NM_001063, GenBank]
*HFE*: Hemochromatosis gene [Reference Sequence NM_139011, GenBank]
MBL: Maternal blood lead
UCBL: Umbilical cord blood lead
μg/dL: Micrograms per deciliter
BPb: Blood lead concentration
CDC: Centers for disease control and prevention, Atlanta, GA
DMT1: Divalent metal transporter 1
TCSS: Tar Creek superfund site
HSPH: Harvard school of public health, Boston, MA
LEAD: Local environmental action demanded agency, Miami, OK
IBRHC: Integris baptist regional health center, Oklahoma City, OK
SNP: Single nucleotide polymorphism
ISO: International Standards Organization
ICP-MS: Inductively-coupled plasma mass spectrometer
NIST SRM: National institute of standards and technology standard reference Material
LOD: Limit of detection
DNA: Deoxyribonucleic acid
TE buffer: A solution used in molecular biology to protect DNA or RNA from degradation, it contains Tris, a common pH buffer, and EDTA, a chelating agent
MALDI-TOF: Matrix-assisted laser desorption ionization – time of flight
*ALAD*: Delta-aminolevulinic acid dehydratase gene
*VDR*: Vitamin D receptor gene.
