# Supplementary material for: Maternal iron metabolism gene variants modify umbilical cord blood lead levels by gene-environment interaction: a birth cohort study
Source: Environ Health. 2014 Oct 6;13:77. doi: 10.1186/1476-069X-13-77 (PMC4271345; doi:10.1186/1476-069X-13-77)
Supplement: Supplementary file 1 — Additional file 1: Parameter estimates from a multivariable regression model evaluating the effects of infant iron metabolism gene variants, log-transformed maternal blood lead, and their interaction on log-transformed umbilical cord blood lead. (PDF 157 KB) [file 12940_2014_803_MOESM1_ESM.pdf]

Additional File 1: Parameter estimates from a multivariable regression model evaluating the effects of *infant* iron metabolism gene variants, log-transformed maternal blood lead, and their interaction on log-transformed umbilical cord blood lead.[a, b, c]

Predictor	β	95% CI	P-value
Constant	-0.17	-0.20, -0.13	**<0.01**
Maternal Blood Lead (MBL)	0.89	0.78, 0.99	**<0.01**
Infant *TF P570S*	-0.05	-0.11, 0.01	0.10
MBL x Infant *TF P570S*	-0.09	-0.28, 0.10	0.34
Infant *HFE H63D*	-0.03	-0.09, 0.03	0.32
MBL x Infant *HFE H63D*	0.02	-0.16, 0.19	0.86
Infant *HFE C282Y*	-0.02	-0.10, 0.06	0.63
MBL x Infant *HFE C282Y*	-0.12	-0.34, 0.09	0.26

[a] Blood lead concentrations are log-transformed, beta coefficients are therefore interpreted as percent change in umbilical cord blood lead for a 1% change in maternal blood lead; [b] Collinearity diagnostics indicate a maximum variance inflation factor of 2.2; [c] bolded values are statistically significant at α = 0.05.
