# Supplementary material for: Maternal iron metabolism gene variants modify umbilical cord blood lead levels by gene-environment interaction: a birth cohort study
Source: Environ Health. 2014 Oct 6;13:77. doi: 10.1186/1476-069X-13-77 (PMC4271345; doi:10.1186/1476-069X-13-77)
Supplement: Supplementary file 2 — Additional file 2: Parameter estimates from a multivariable regression model evaluating the effects of maternal iron metabolism gene variants, log-transformed maternal blood lead, and their interaction on log-transformed umbilical cord blood lead. (PDF 72 KB) [file 12940_2014_803_MOESM2_ESM.pdf]

Additional File 2: Parameter estimates from a multivariable regression model evaluating the effects of *maternal* iron metabolism gene variants, log-transformed maternal blood lead, and their interaction on log-transformed umbilical cord blood lead.[a, b, c]

Predictor	β	95% CI	P-value
Constant	-0.18	-0.21, -0.15	**<0.01**
Maternal Blood Lead (MBL)	0.93	0.83, 1.02	**<0.01**
Maternal *TF P570S*	0.01	-0.05, 0.07	0.75
MBL x Maternal *TF P570S*	-0.13	-0.33, 0.06	0.18
Maternal *HFE H63D*	-0.06	-0.12, -0.01	**0.05**
MBL x Maternal *HFE H63D*	-0.05	-0.23, 0.14	0.60
Maternal *HFE C282Y*	-0.02	-0.10, 0.07	0.72
MBL x Maternal *HFE C282Y*	-0.29	-0.52, -0.05	**0.02**

[a] Blood lead concentrations are log-transformed, beta coefficients are therefore interpreted as percent change in umbilical cord blood lead for a 1% change in maternal blood lead; [b] Collinearity diagnostics indicate a maximum variance inflation factor of 2.1; [c] bolded values are statistically significant at $\alpha = 0.05$.
